# Assessing lower extremity loading during activities of daily living using continuous-scale physical functional performance 10 and wireless sensor insoles: a comparative study between younger and older adults

**DOI:** 10.1007/s00068-023-02331-8

**Published:** 2023-07-22

**Authors:** Sonja Häckel, Tobias Kämpf, Heiner Baur, Arlene von Aesch, Reto Werner Kressig, Andreas Ernst Stuck, Johannes Dominik Bastian

**Affiliations:** 1 Department of Orthopaedic Surgery and Traumatology, Inselspital, University Hospital Bern, University of Bern, Freiburgstrasse 18, 3010 Bern, Switzerland; 2 Department of Health Professions, Bern University of Applied Sciences, Murtenstrasse 10, Bern, Switzerland; 3 Physiotherapie SportClinic Zurich, Giesshübelstrasse 15, 8045 Zurich, Switzerland; 4 University Department of Geriatric Medicine Felix Platter and University of Basel, Basel, Switzerland; 5 Department of Geriatrics, Inselspital, Bern University Hospital, University of Bern, Bern, Switzerland

**Keywords:** Weight-bearing, Lower extremity, Physical functional performance, Wireless sensor insoles, Old, Young adult

## Abstract

**Purpose:**

This study aims to investigate the lower extremity loading during activities of daily living (ADLs) using the Continuous Scale of Physical Functional Performance (CS-PFP 10) test and wireless sensor insoles in healthy volunteers.

**Methods:**

In this study, 42 participants were recruited, consisting of 21 healthy older adults (mean age 69.6 ± 4.6 years) and 21 younger healthy adults (mean age 23.6 ± 1.8 years). The performance of the subjects during ADLs was assessed using the CS-PFP 10 test, which comprised 10 tasks. The lower extremity loading was measured using wireless sensor insoles (OpenGo, Moticon, Munich, Germany) during the CS-PFP 10 test, which enabled the measurement of ground reaction forces, including the mean and maximum total forces during the stance phase, expressed in units of body weight (BW).

**Results:**

The total CS-PFP 10 score was significantly lower in older participants compared to the younger group (mean total score of 57.1 ± 9.0 compared to 78.2 ± 5.4, respectively). No significant differences in the mean total forces were found between older and young participants. The highest maximum total forces were observed during the tasks ‘endurance walk’ (young: 1.97 ± 0.34 BW, old: 1.70 ± 0.43 BW) and ‘climbing stairs’ (young: 1.65** ± **0.36 BW, old: 1.52 ± 0.28 BW). Only in the endurance walk, older participants showed a significantly higher maximum total force (*p* < 0.001).

**Conclusion:**

The use of wireless sensor insoles in a laboratory setting can effectively measure the load on the lower extremities during ADLs. These findings could offer valuable insights for developing tailored recommendations for patients with partial weight-bearing restrictions.

## Introduction

The optimal weight-bearing and mobilization protocol for postoperative rehabilitation of the lower extremity, especially in patients with fragility fractures, has been a topic of ongoing debate. While some experts suggest protocols that involve immediate mobilization and weight-bearing, others argue for more cautious approaches, such as bed-to-wheelchair mobilization. However, limited evidence exists to conclusively determine which approach is superior [1, 2]. A contributing factor to this controversy may be the limited knowledge about the exact load placed on the leg during activities of daily living (ADL). While previous research has focused on measuring weight-bearing using methods such as force plates, pressure sensors, and motion capture systems [3–5], these have primarily been limited to static situations, such as standing or sitting still, and laboratory settings. The utilization of wireless sensor insoles to measure ground reaction forces (GRFs) during ADLs represents a promising new approach for gaining a better understanding of lower extremity loading in ADLs. These insoles are equipped with capacitive pressure sensors that measure plantar pressures. This ensures the calculation of applied ground reaction forces during stance and provides simple spatiotemporal gait parameters [6]. Some studies have utilized wireless sensor insoles to measure weight bearing during specific activities, such as walking or stair climbing, but there is limited research that has used these sensors to measure weight bearing during a broader range of activities [7, 8].

The Continuous-Scale Physical Functional Performance (CS-PFP) test offers the possibility to assess an individual's physical performance [9]. The short form of the test, the CS-PFP 10, includes 10 tasks that evaluate various aspects of functional mobility such as stair climbing, carrying groceries, and walking on different surfaces [10]. The present study aimed to investigate GRFs of the lower extremity during ADLs using the CS-PFP 10 test and wireless sensor insoles.

## Methods

### Study design and participants

This study utilized a prospective comparative cohort design and received a waiver of ethical approval from the Institutional Review Board (IRB). Two groups were formed: one group included female and male ambulatory volunteers aged 65 years or older, without either a lack of motivation, frailty, impaired cognition, gait disturbances, or previous orthopedic surgery on the lower leg. The other group included volunteers under 30 years of age. To assess motivation, the short version of the “Geriatric Depression Scale-15 (GDS)” [11] was used, and cognition was evaluated using the “Montreal Cognitive Assessment (MoCA-Score)” [12] and “Mini-Mental State (MMS) [13].” Grip strength was measured to test for weakness [14]. The “FRAIL scale” [15] “Katz Index,” [16], and “SARC-F” [17] were collected to describe the functional status and frailty of the participants. The study included 42 participants who met the following inclusion criteria: Age < 30 years or > 65 years, no musculoskeletal limitations, independence in ADLs without assistance from others (as self-reported), and self-consideration as fit. The exclusion criteria were individuals with gait disorders, frailty, or cognitive impairments.

Between the two groups, significantly different grip strength and FRAIL scale were seen (Table 1).

**Table 1:** Demographic characteristics and clinical scores of the study participants

Participants characteristics	Young adults (*n* = 21)	Older adults (*n* = 21)	*p* value
Female, *n* (%)	10 (47.6)	12 (57.1)	0.792
Age (years), mean (SD)	23.6 (± 1.8)	69.6 (± 4.6)	< 0.001
Height (cm), mean (SD)	178.4 (± 10.7)	172.5 (± 9.8)	0.274
Weight (kg), mean (SD)	72.9 (± 15.1)	74.8 (± 15.0)	0.791
BMI, mean (SD)	22.7 (± 2.8)	24.9 (± 3.4)	0.123
Motivation			
GDS-15, mean (SD)	0 (± 0)	0.15 (± 0.7)	> 0.999
Pathological result ≥ 5 (*n*)	**0**	**0**	
Cognition			
MoCA-Score, mean (SD)	29.5 (± 0.6)	28.7 (± 1.2)	0.094
Pathological result < 26 (*n*)	**0**	**0**	
MMS, mean (SD)	29.8 (± 0.4)	29.4 (± 0.6)	0.282
Pathological result < 24 (*n*)	**0**	**0**	
Weakness			
Handgrip strength (kg), mean (SD)	38 (± 11)	30 (± 8)	0.114
Handgrip strength (*N*), mean (SD)	377 (± 108)	293 (± 80)	0.114
Pathological result (*n*) [11]	**7**	**5**	
Frailty			
FRAIL Scale, mean (SD)	1.2 (± 0.4)	1.9 (± 0.4)	< 0.001
Pathological result ≥ 3 pt. (*n*)	**0**	**0**	
Katz Index, mean (SD)	6 (± 0)	6 (± 0)	> 0.999
Pathological result < 6 (*n*)	**0**	**0**	
SARC-F, mean (SD)	0 (± 0)	0.2 (± 0.4)	0.286
Pathological result > 4 pt. (*n*)	**0**	**0**	

Geriatric Depression Scale-15 (GDS-15), Montreal Cognitive Assessment (MoCA), and Mini-Mental State (MMS) scores are presented. Nonparametric tests (Mann–Whitney test) with *p* values adjusted using the Holm–Šídák method were used for statistical analysis

*p* < 0.05 is considered statistically significant (in bold)

### Standardized measurement of ADLs

The CS-PFP-10 test was employed as a standardized measurement for assessing ADLs. The CS-PFP-10 test consists of ten tasks (Table 2), which assess the physical functioning of subjects across five subgroups: upper body strength (UBS), lower body strength (LBS), upper body flexibility (UBF), balance and coordination (BAC), and endurance (END) [9, 10]. The test is scored on a point scale and includes all abilities relevant to ADLs. The tasks gradually increase in intensity, from test situation to test situation (1–10), to accurately measure the physical abilities of the subjects. Participants are instructed to perform each task with maximum effort, completing them as quickly as possible and carrying as much weight as possible. To ensure safety, a safety belt is worn by the participant, allowing the tester to catch any potential falls. Additionally, subjects are closely monitored during all tasks (Table 2). The CS-PFP 10 total score is obtained by calculating the average corrected score of all tasks, while the total score for each domain is obtained by calculating the average score of the tasks in that domain. The scoring system for the CS-PFP 10 test ranges from 0 to 100, where a score of 0 to 47 indicates an increased likelihood of functional dependence, a score of 48 to 56 indicates being at risk of losing independence, and a score of 57 to 100 predicts independence in ADLs [10, 18].

**Table 2:** Overview of the Continuous Scale of Physical Functional Performance (CS-PFP 10) task

Task	Description	Task effort	Tested subgroups	Measurements
1	Kitchen pot carry	Low effort (personal)	Upper body strength, balance, and coordination	Time, weight
2	Put on/take off a jacket		Upper body flexibility, balance, and coordination	Time
3	Scarves pickup		Lower body strength, balance, and coordination	Time
4	Maximal reach		Upper body flexibility, balance, and coordination	Distance (initial and final)
5	Floor sweep	Medium effort (household)	Lower body strength, balance, and coordination	Time
6a	Laundry loading		Upper body strength, Lower body strength	Time
6b	Laundry unloading		Upper body strength, Lower body strength	Time
7	Sit down and get up from the floor		Lower body strength, balance, and coordination	Time
8	Stair climbing	Hard effort (mobility)	Lower body strength	Time
9	Grocery carrying and walking		Upper body strength, Lower body strength, balance, and coordination	Time and weight
10	6 min walk		Endurance	Distance

### Load measurement of the lower extremity

**Fig. 1:**
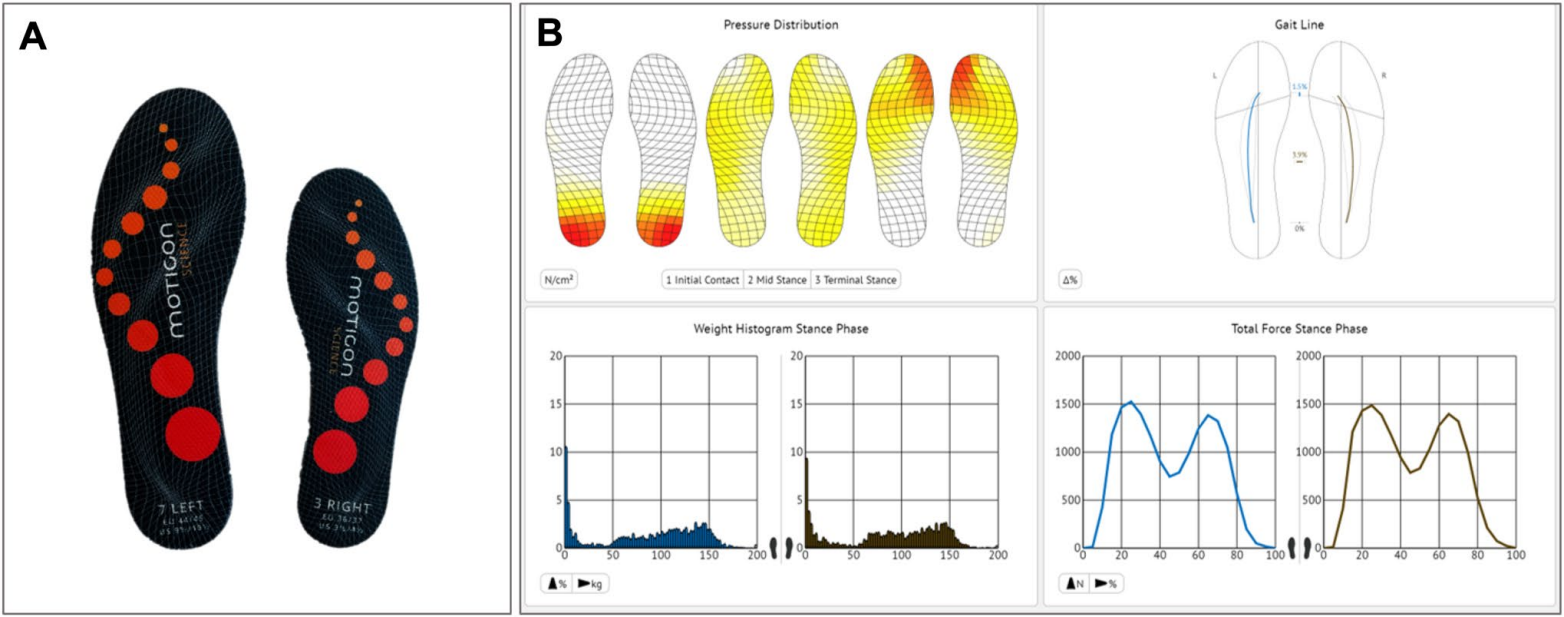
**A** Picture of the wireless OpenGo Sensor Insoles (Moticon, Germany). **B** Example of software analysis (screenshot)

The primary outcome of this study was the maximum total force, which refers to the highest amount of force exerted on the ground during the stance phase expressed in units of body weight (BW). Secondary outcomes were the mean total force, which refers to the average amount of force exerted on the ground, expressed in BW [3], and the CS-PFP 10 score of the participants. To measure the maximum and mean, we used the OpenGo insole (Moticon GmbH, Munich, Germany) containing 13 capacitive pressure sensors and a 3D accelerometer (Fig. 1A) [19]. The insole was placed inside the participants’ shoes, and it measured the force exerted during the tasks of the CS-PFP-10. The proprietary Moticon science software (Version 03.03.20) (Fig. 1B) automatically analyzed and calculated the mean and maximum ground reaction forces.

### Experimental protocol

The recruitment of participants was initiated according to the aforementioned inclusion criteria. Additional scores were collected following participants’ consent to participate in the study. Furthermore, all participants received instructions based on the guidelines provided for the CS-PFP-10 test. Subsequently, all participants were equipped with the appropriate sensor insoles and commenced the CS-PFP-10 test. Once the test was completed, the CS-PFP-10 scores were calculated, and the recorded GRFs were analyzed using specialized software (Moticon, Germany).

### Statistical Analysis

For non-normally distributed continuous data, a nonparametric Mann–Whitney test with p-values adjusted using the Holm–Šídák method was used for analysis. Normally distributed continuous data were analyzed using ordinary two-way ANOVA. Data are presented as mean ± SD, and the level of significance was set at *p* < 0.05. *p* values were calculated with a 95% confidence interval using IBM SPSS Statistics Version 28.0 for Macintosh (IBM Corp., Armonk, NY, USA).

## Results

**Fig. 2:**
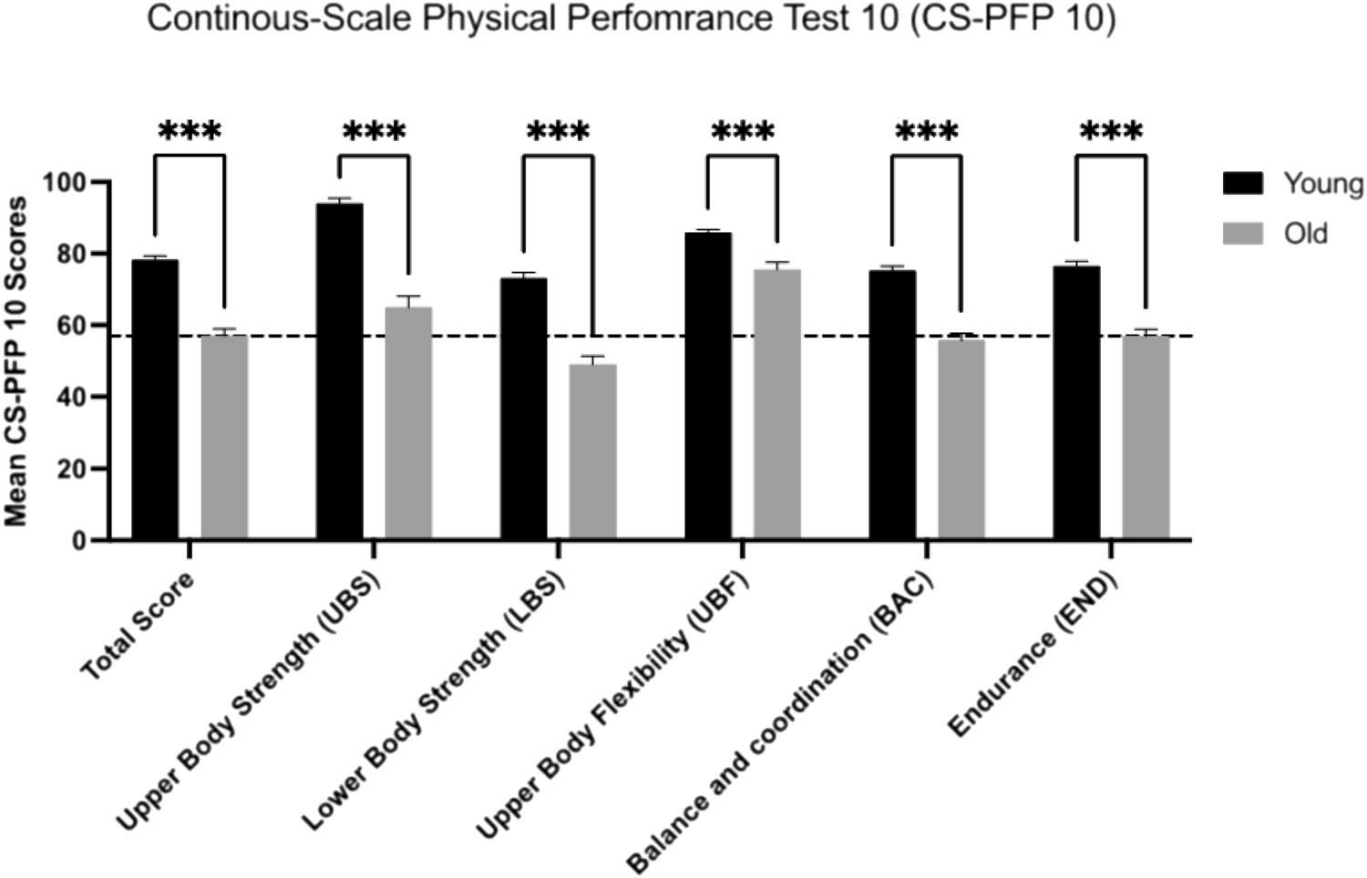
Continuous-Scale Physical Performance Test 10 (CS-PFP 10) Results in Young and Older Participants. The figure displays the performance scores of young and old participants on the 10 tasks of the CS-PFP 10 test, categorized into five subcategories. Young volunteers scored significantly higher than old participants in all subcategories. The dotted line represents the score threshold of ≥ 57 points, indicating a physical reserve and independent living status. Statistical significance was determined using ordinary two-way ANOVA with ***indicating *p* < 0.001

The analysis of the PFP-10 test showed that the group with younger participants had significantly higher scores compared to the older group, with a mean total score of 78.2 (SD 5.4) compared to 57.1 (SD 9.0), respectively (Fig. 1). The UBS subscore was highest in the young group (94.0, SD 6.6), while the older group did best in UBF (75.6 SD 9.4). Both groups had the lowest subscore in LBS, with the younger group scoring 73.2 (SD 7.3) and the older group significantly lower at 49.1 (SD 10.7) (Fig. 2).

Three older participants in our study scored below 47 on the CS-PFP 10 test, indicating dependence on activities of daily living. Nevertheless, we included them in our analysis of lower extremity loading as they reported being independent in ADLs, with no assistance required from third parties.

### Lower extremities loading during activities of daily living

Ground reaction forces on each limb were evaluated using the wireless OpenGo insole (Moticon GmbH, Munich, Germany) in all 10 tasks of the CS-PFP-10. However, due to a measurement error, only 20 older participants were included in the analysis.

**Fig. 3:**
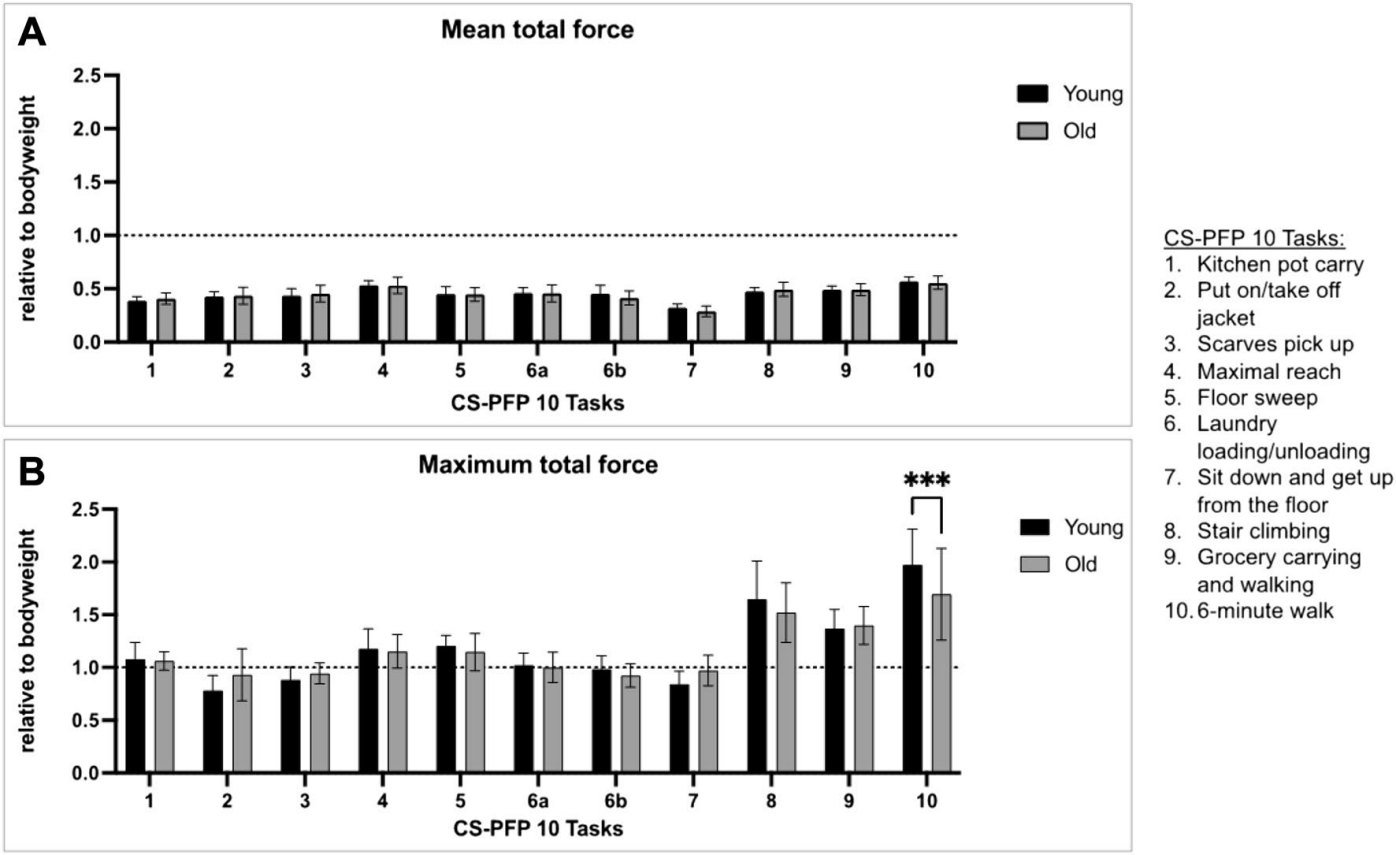
Comparison of ground reaction forces during the CS-PFP-10 test (10 tasks) between young (*n* = 21) and old healthy (*n* = 20) volunteers.** A** Mean total force and **B** Maximum total force were measured using OpenGo Sensor Insoles and are presented in units of body weight. The young group had a significantly higher maximum total force, but there were no significant differences between the two groups in mean total force. The data shown are the means of measurements from both right and left insoles. Statistical significance was determined using ordinary two-way ANOVA, with ***indicating *p* < 0.001

In terms of the maximum total force, younger participants showed significantly higher forces in Task 10 (6-min walk), with *p* < 0.001. The hard effort tasks of the CS-PFP 10, including Task 8 (stair climbing), Task 9 (grocery carrying and walking), and Task 10 (6-min walk), exhibited the highest maximum force of 1.37 ± 0.18 BW to 1.97 ± 0.34 BW in younger participants and 1.40 ± 0.18 BW to 1.70 ± 0.43 BW in older participants (Fig. 3B, Table 3).

**Table 3:** Comparison of ground reaction forces during the CS-PFP-10 test (10 tasks) between young (*n* = 21) and old healthy (*n* = 20) volunteers

Mean total force (unit to body weight), SD				Maximum total force (unit to bodyweight), SD			
CS-PFP 10 task	Young adults	CS-PFP 10 task	Older adults	CS-PFP 10 Task	Young adults	CS-PFP 10 task	Older adults
10	0.57 ± 0.04	**10**	0.56 ± 0.06	**10**	1.97 ± 0.34	**10**	1.70 ± 0.43
4	0.53 ± 0.04	**4**	0.53 ± 0.08	**8**	1.65 ± 0.36	**8**	1.52 ± 0.28
9	0.49 ± 0.04	**8**	0.49 ± 0.07	**9**	1.37 ± 0.18	**9**	1.40 ± 0.18
8	0.47** ± **0.04	**9**	0.49** ± **0.06	**5**	1.20** ± **0.10	**4**	1.15** ± **0.16
6a	0.46** ± **0.05	**6a**	0.46** ± **0.08	**4**	1.18** ± **0.19	**5**	1.15** ± **0.18
6b	0.45** ± **0.08	**3**	0.45** ± **0.08	**1**	1.08** ± **0.16	**1**	1.06** ± **0.09
5	0.45** ± **0.07	**5**	0.45** ± **0.06	**6a**	1.02** ± **0.11	**6a**	1.00** ± **0.14
3	0.44** ± **0.06	**2**	0.43** ± **0.08	**6b**	0.98** ± **0.13	**7**	0.97** ± **0.14
2	0.43 ± 0.04	**6b**	0.41 ± 0.07	**3**	0.88 ± 0.12	**3**	0.95 ± 0.10
1	0.39 ± 0.04	**1**	0.41 ± 0.05	**7**	0.84 ± 0.12	**2**	0.93 ± 0.25
7	0.32 ± 0.04	**7**	0.29 ± 0.05	**2**	0.78 ± 0.14	**6b**	0.92 ± 0.11

Mean total force and maximum total force were measured using OpenGo Sensor Insoles and are presented in units of body weight, sorted from highest to lowest force. Highlighted in underline are the three highest and lowest tasks. The data shown are the means of measurements from both right and left insoles

*p* < 0.05 is considered statistically significant (in bold)

Comparing young (*n* = 21) and older (*n* = 20) participants, there was no significant difference in the mean total force (Fig. 3). The highest mean total force for both age groups was achieved in Task 10 (6-min walk), with 0.57 ± 0.04 BW and 0.56 ± 0.06 BW for young and older participants, respectively. The lowest force was observed in Task 7 (sit down and get up from the floor), with 0.32 ± 0.04 BW and 0.29 ± 0.05 BW for young and older participants, respectively (Fig. 3A, Table 3).

To investigate the impact of older participants' independence in ADLs based on their CS-PFP 10 score, we categorized them into three subgroups: dependent (*n* = 3), borderline independent (*n* = 8), and independent (*n* = 9). Subsequently, we analyzed the GRFs for each task of the CS-PFP 10 test within each subgroup and found no significant differences in the mean or maximum total forces relative to BW (data not shown).

## Discussion

This study aimed to use the CS PFP-10 test to compare the lower extremity force during ADLs between healthy young and older participants. This study is the first to investigate the lower extremity load during ADLs in both age groups using the CS-PFP 10 test.

There is currently a lack of a systematic approach to personalizing postoperative rehabilitation protocols for old patients after fracture fixation, as discussed in previous research [2]. The ideal rehabilitation protocol should balance the need to protect those who cannot comply with weight-bearing restrictions while also assisting others in mobilization with partial weight-bearing and minimizing the risk of inadvertent overloading. In clinical practice, various considerations influence surgeons when determining weight-bearing restrictions in older adults. These factors include fracture type, comminution, bone quality, the accuracy of reduction, implant positioning and stability, as well as patient-specific factors such as the ability to adhere to postoperative weight-bearing restrictions. There is also a potential “cost–benefit ratio” to consider when deciding on these restrictions, particularly in patients who are unable to comply with partial weight-bearing instructions [20]. Overloading the osteosynthetic construct before fracture healing may result in failure, requiring revision surgery with significant morbidity in older patients. Unstable trochanteric fractures, especially in the presence of poor bone quality and suboptimal fracture fixation, have been associated with failure rates of over 50% [21]. Accordingly, no clear consensus exists on optimal aftercare for unstable trochanteric fractures treated with intramedullary nailing [2]. On the other hand, prolonged immobilization can have several detrimental effects. It can lead to muscle disuse and atrophy, resulting in muscle weakness, loss of muscle mass, and decreased functional capacity [22]. Immobilization can also cause joint stiffness and contractures, limiting the range of motion and impairing joint function, leading to decreased mobility and difficulties with ADLs [23]. Additionally, immobilization can accelerate bone loss, increasing the risk of osteoporosis and fractures, further compromising the healing process and functional recovery [24]. Therefore, early weight bearing and minimizing immobilization are beneficial to preserve muscle strength, joint function, bone density, and overall functional recovery in older adults.

The CS-PFP-10 is a widely accepted and validated tool for assessing an individual's ability to perform ADLs. Its tasks are standardized with precise specifications, making it a valuable tool for cross-laboratory comparisons. Previous studies have utilized the CS-PFP-10 to evaluate patients with various medical conditions, including chronic obstructive pulmonary disease and heart failure, as well as to investigate the relationship between functional performance and the risk of falls [25–27]. In our study, all young participants scored > 57, indicative of physical reserve and independent living status [28]. The results of our study demonstrate that older participants had significantly lower scores on both the total CS-PFP 10 score and all subcategories compared to their younger counterparts. Notably, three older participants scored below 47 on the CS-PFP 10 test, indicating a lower physical reserve and a dependent living status. Due to their self-reported independence, we included them in our analysis. We acknowledge that the use of subjective self-reported values rather than objective measurement tools such as the Short-Form Health Survey Physical Function scale may have limitations. Nevertheless, other measurements of cognitive function, weakness, and frailty did not reveal any significant difference between younger and older participants.

To accurately measure load bearing during ADLs, we utilized a wireless sensor insole (Open Go, Moticon, Germany), which has already been clinically validated [6]. This innovative technology has been utilized in various clinical studies, including those focused on gait analysis in older patients and also in Parkinson's patients [29] as well as analyzing pathological gait patterns after talus fractures [30]. Studies have shown that using sensors and providing biofeedback can improve adherence to weight-bearing restrictions [31, 32]. This adherence is crucial for successful post-surgical outcomes. Additionally, wireless sensor insoles can be particularly useful for patients in remote areas, allowing healthcare professionals to monitor adherence to weight-bearing instructions in telehealth settings [33]. To the best of our knowledge, this is the first study to use this technology for measurements of ADLs. In this study, we specifically directed our attention to ADLs rather than focusing on exercises typically performed in a rehabilitation setting. The rationale behind this choice was to investigate ADLs in order to refine post-surgical protocols not only within a rehabilitation environment but also in the patients' home and daily lives. Exercises often involve higher magnitudes of load and repetitive loading patterns compared to ADLs [34]. These exercises are designed to intentionally apply controlled loads to target specific muscle groups or achieve specific fitness goals. On the other hand, ADLs encompass a broader range of movements and loading patterns that may be less predictable or repetitive in nature.

Our results identified two tasks with a high load on the lower limb (> 1.5 BW) in all participants, regardless of age. Stair climbing (CS-PFP 10 task 9) and a 6-min walk (CS-PFP 10 task 10) resulted in the highest loads on the lower limb. We found a significant difference in maximum total forces between younger and older participants in the 6-min walk test (CS-PFP 10 task 10). However, there was no significant difference in the mean total force on the lower extremity load. The literature suggests that older individuals tend to adopt a more cautious gait [35]. Other studies have shown that age-related decline in muscular capabilities at the ankle may contribute to decreased walking performance in older adults [36]. Moreover, reduced overall muscular strength could negatively affect gait performance [37].

### Limitations and Strengths

One potential limitation of the CS-PFP 10 test is its duration, which takes approximately one hour per subject and requires a certified examiner to be present throughout the test. While this standardized approach is a strength, it may be taxing for participants and examiners alike. However, the reliability and validity of the CS-PFP 10 test have been established in previous studies. Using the validated German version in this study further strengthens its utility for measuring physical function in older adults [18, 28]. Another limitation is that the GDS, MoCA, MMS, FRAIL Scale, Katz Index, and SARC-F used in our study have been primarily validated in older adults and may not have the same level of applicability or accuracy when used in young individuals. However, for the purpose of our study, these scores were utilized to characterize the daily functioning of both young and older participants.

A strength of the CS-PFP 10 test is its ability to assess the performance of healthy individuals and identify specific areas of weakness in strength, balance, flexibility, and endurance. Other tests, like the Short Physical Performance Battery or the Physical Performance Test, may not detect these differences due to a ceiling effect in physically healthier and active individuals [38]. However, future research should also examine the test's efficacy in a postoperative setting with various walking aids and weight-bearing restrictions. Additionally, for individuals using assistive devices. While various tasks, such as putting on a jacket, picking up scarves from the floor, or walking for 6 min, could be done using crutches or a walker, some tests may need to be modified. Modifications of the CS-PFP 10 have already been made to accommodate people in wheelchairs with the WC-PFP test [39]. On the other hand, the assessment of lower extremity loading during the CS-PFP-10 test may provide insight into the level of load placed on the leg for non-compliant patients who do not use prescribed assistive devices such as crutches or walkers at home.

## Conclusion

In conclusion, the utilization of the sensor insole provided valuable insight into the accurate and reliable measurement of lower extremity loading during various ADLs. The study results emphasize the significance of recognizing the high load on lower extremities during ADLs, regardless of age. For patients with weight-bearing restrictions, tasks such as stair climbing, and endurance walking require special attention due to the high loading. Future studies should investigate the impact of specific comorbidities on lower extremity force during ADLs in older adults. Overall, these findings highlight the potential for this technology to be used in clinical settings to evaluate lower extremity loading during ADLs and develop targeted interventions to improve physical function and independence.

## Data Availability

The data that support the findings of this study are available from the corresponding author upon reasonable request. The data are not publicly available due to privacy or ethical restrictions. However, the data can be made request. The data are not publicly available due to privacy or ethical restrictions. However, the data can be made available for research purposes with appropriate permissions and approvals from the relevant institutional review boards. Researchers interested in accessing the data should contact the corresponding author to discuss the terms and conditions for data sharing.
